# Novel Quantification of Eggshell Surfaces in *Dromaius novaehollandiae* With Implications for the Fossil Eggshells of Oviraptorosauria (Dinosauria)

**DOI:** 10.1002/ece3.71260

**Published:** 2025-05-05

**Authors:** Joshua Hedge, Emerald Bender, Lindsay E. Zanno

**Affiliations:** ^1^ Department of Biology North Carolina State University Raleigh North Carolina USA; ^2^ Paleontology North Carolina Museum of Natural Sciences Raleigh North Carolina USA; ^3^ Department of Earth Sciences Montana State University Bozeman Montana USA

**Keywords:** bird, eggshell, orientation, ornamentation, quantitative, theropod

## Abstract

The external surfaces of non‐avian dinosaur eggs are not usually smooth like those of their avian descendants. Unique ornamentation patterns sculpt the exterior of the eggs, a trait that is difficult to interpret because of its scarcity in modern taxa. One avian species that does homoplastically present similar external eggshell ornamentation to that of non‐avian dinosaurs is *Dromaius novaehollandiae* Latham, 1790, the emu. Here we use *D. novaehollandiae* eggs in conjunction with a clutch of oviraptorosaurian dinosaur eggs (NCSM 33576, *Macroelongatoolithus carlylei*) to test new methods of quantifying external eggshell ornamentation. Currently, the only scientific language for describing and comparing ornamentation styles in fossil ootaxa is restricted to qualitative categorization, which introduces issues of subjectivity and overly broad and overlapping typification. In this study, we derived and tested a new statistical quantitative approach to quantifying ornamentation that includes two existing functions of the molaR package in R previously applied to shape quantifications of fossil teeth, and ‘Orientation’, a novel function presented as a proxy for ‘direction’, needed to capture directionality. Results demonstrate that (1) the quantitative approach provides statistical backing to gross qualitative observations; (2) statistically significant differences exist between the ornamentation in *D. novaehollandiae* and *M. carlylei*, particularly in terms of relief; (3) intranest variation of *M. carlylei* can be demonstrated from harmonic mean *p*‐value differences between different pairs of eggs. This method offers a strong platform to consolidate quantitative measures with existing qualitative categories, improve the diagnoses of ootaxa and answer broad ecological and evolutionary questions regarding dinosaur reproduction. Moreover, wider application of the technique is encouraged for a multi‐proxy quantitative analysis of any paleontological surfaces, such as echinoderm tests, geological ripple marks or dentition.

## Graphical abstract

We have developed a new, quantitative method of assessing the variation on paleosurfaces. This method uses functions from the molaR package of R, including new functions detailed here. This paper tests the functions on the eggshell surfaces of extant emu ( *Dromaius novaehollandiae* ) and fossil oviraptorosaur eggs of the ootaxon *Macroelongatoolithus carlylei*.

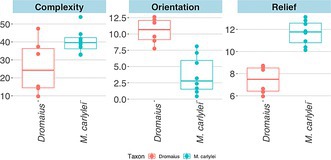

## 1 Introduction

The external surface of emu ( *Dromaius novaehollandiae* ) eggs is unique in the modern avian world. The large, blue‐to‐green eggs of this paleognath bird are sculpted into an array of nodes and ridges designated as ornamentation. This ornamentation is remarkable for its complexity compared to other birds—comparable only to the eggs of cassowaries (*Casuarius*)—and for the variation observable on gross inspection. This variation in ornamentation is visually detectable intraspecifically and within a single nesting population.

No true homolog of the external ornamentation of *D. novaehollandiae* eggs exists in extant taxa or the fossil record. Non‐avian theropod dinosaurs predominantly have two‐layered eggshells consisting of an inner mammillary layer and an outer crystalline layer (Hirsch and Quinn 1990; Mikhailov et al. 1996), whereas earlier‐diverging dinosaur lineages, including hadrosaurs (Barta), sauropods (Grellet‐Tinner et al. 2004) and some non‐maniraptoran theropods (*Torvosaurus* (Araújo et al. 2013) and *Lourinhanosaurus* (Russo et al. 2017)) and modern crocodiles (Mikhailov et al. 1996; although see Moreno‐Azanza et al. 2015) have a single‐layered eggshell. The ornamentation seen in most non‐avian dinosaurs manifests as an enhancement to this second layer—the continuous or crystalline layer—that sculpts nodes, ridges or a system of both on the external surface. Birds and some close relatives among non‐avian theropods, the troodontidae ootaxa *Prismatoolithus levis* (Jackson et al. 2010) and *Prismatoolithus ilekensis* (Skutschas et al. 2017) have a three‐layered eggshell. The eggs of the paleognath bird *D. novaehollandiae* exhibit a manipulation only of the third outermost layer—the external layer—into similar patterns, or a fourth layer that is exclusively the ornamentation (Grellet‐Tinner 2006). Throughout modern Avialae, the external layer is modified through color alteration or maculation (speckling) by pigments, but in rarer cases like the *D. novaehollandiae* , the structure of the shell itself is physically altered, analogous to that of some non‐avian dinosaurs. Fossil eggs of extinct emu that have smooth external eggshells (Williams 1981) offer evidence that modern *D. novaehollandiae* eggshell ornamentation evolved independently. This is probably an alternative strategy to cuticle seen in other paleognath birds in protecting pores that are situated underneath nodes and ridges of ornamentation in *D. novaehollandiae* (Board and Tullett 1975) from microorganism infiltration (Szczerbińska and Wiercińska 2014).

Among extant taxa, *D. novaehollandiae* are perhaps the single best‐living analog for nesting in late‐diverging theropod dinosaurs: the eggs are relatively large (*c*. 13 × 9 cm; Campbell and Lack 2013); eggshell thickness compares approximately at 1.1 mm (Williams 1981); the reproductive mode is similar to that of oviraptorosaurs (Hogan 2024) or Troodontidae (Varricchio et al. 2018); they are ground nesters; the eggs are blue and green colored, utilizing high percentages of biliverdin pigment, as has recently been reported in elongatoolithid eggs (Wiemann et al. 2017); and the young hatch precocially with long incubation periods, as has been suggested for maniraptoran dinosaurs (Varricchio et al. 2018). The comparison to *M. carlylei*—within the oofamily Elongatoolithidae—is of particular interest because these are among the largest eggs in the fossil record (Li et al. 1995; Huh et al. 2014; Simon et al. 2018) with the most elaborate and variable ornamentation.

Since the discovery of fossil eggs, scientists have looked for methods to categorize them. However, only in the 1970s were fossil eggs categorized in a fashion akin to body fossils, when Zhao (1975) introduced a parataxonomic system that sorted eggs into ootaxa comprised of oofamilies, oogenera and oospecies. This system remained only regionally known until the system was translated into English, presented more widely and standardized in the 1990s (Hirsch and Quinn 1990; Mikhailov et al. 1996; Carpenter 1999). This system includes both gross morphological and microstructural criteria, now more readily testable with technologically advanced imaging techniques, such as micro‐computed tomography (μCT), digital surface scanning and scanning electron microscopy (Carpenter 1999). These microstructural features, usually seen in radial thin section, include the composite layers of the eggshell (e.g., relative thicknesses and relationship of the contact), more precise eggshell thicknesses and the cross‐sectional nature of pores.

**FIGURE 1:**
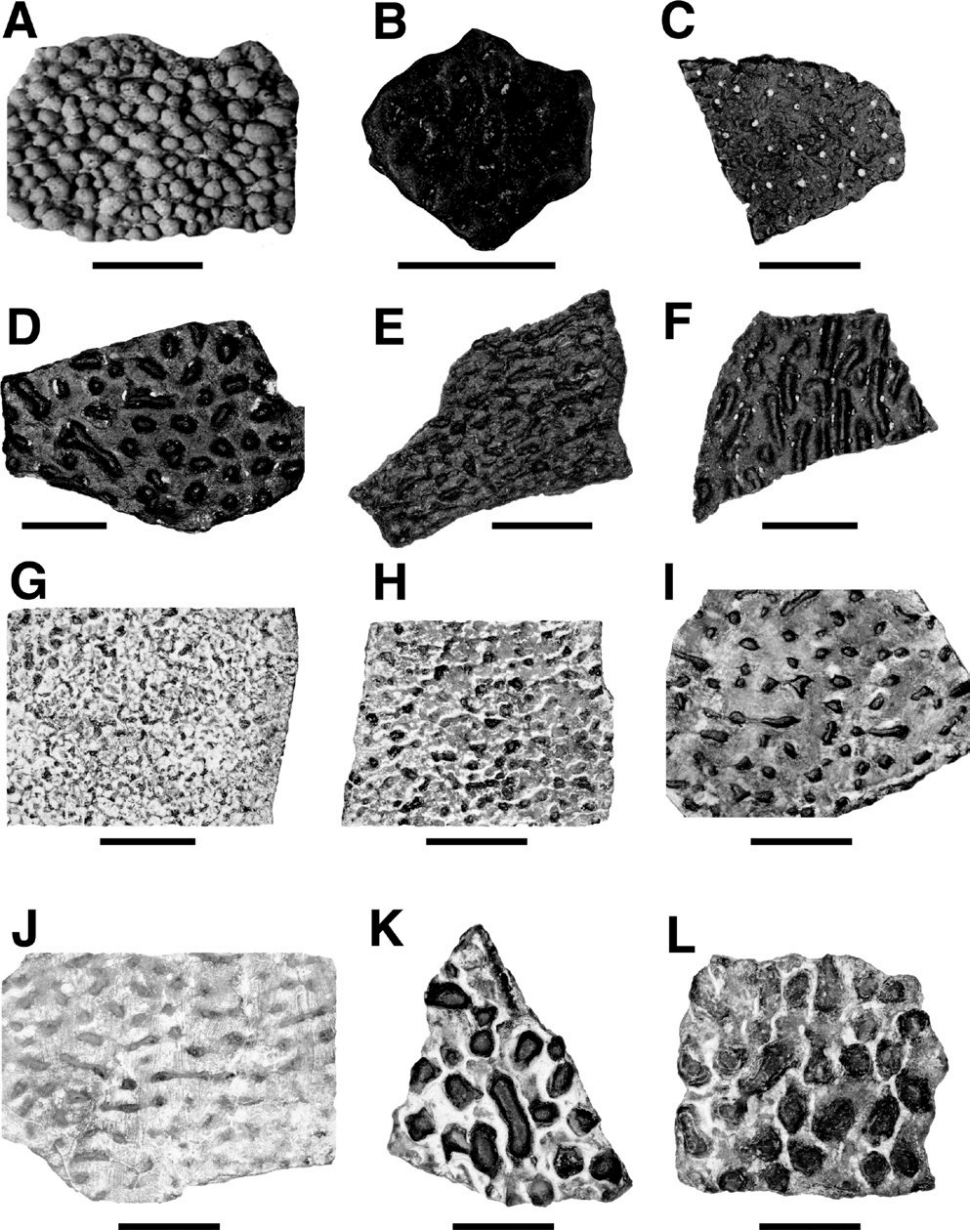
Current ornamentation categorization using binned subjective categories oriented and oriented (Carpenter 1999; Hedge et al. 2023). (A) *Megaloolithidae indet*. from Teel Ulaan Chaltsai (Mongolia) showing unoriented compactituberculate ornamentation. (B) *Macroelongatoolithus carlylei* from the Cedar Mountain Formation of Utah showing unoriented sagenotuberculate ornamentation. (C) *Spheroolithus albertensis* from the Cedar Mountain Formation of Utah showing oriented anastamotuberculate ornamentation. (D) *Continuoolithus canadensis* from the Cedar Mountain Formation of Utah showing unoriented dispersituberculate ornamentation. (E) *Macroelongatoolithus carlylei f*rom the Cedar Mountain Formation of Utah showing oriented ramotuberculate ornamentation. (F) *M. carlylei* from the Cedar Mountain Formation of Utah showing oriented lineartuberculate ornamentation. (G–L), Fragments associated only with the nest NCSM 33576 showing a range of ornamentation types. Scale bars = 5 mm.

In addition, the ornamentation style as seen externally is a criterion used in parataxonomy. This criterion currently consists of six categories of ornamentation (Carpenter 1999) (Table 1) that can be divided into groups that are oriented and unoriented (Hedge et al. 2023) (Figure 1). Although this categorization was effective at the outset of eggshell parataxonomy, it has lost some of its potency. The number of ootaxa currently in the literature is drastically higher; where 29 dinosaur‐affiliated oogenera are named in (Carpenter 1999), there are now at least 70 in the literature with confident dinosaur affiliation (Fernández and Khosla 2015; Kim et al. 2019; Tanaka et al. 2020; Choi et al. 2022; Kundrát and Cruickshank 2022; Zhu et al. 2022), more oofamilies and a greater number of oospecies within these. Moreover, the ootaxonomic quality of these ornamentation bins is hindered by eggs that exhibit more than one of the categories, which have not been updated or expanded since their inception. Ootaxa within Elongatoolithidae can exhibit up to three of the six ornamentation categories at multiple hierarchies within nests, pairs of eggs and on isolated eggs. The terms are also inconsistently applied, due to increasingly nebulous boundaries between ornamentation types observed in newly discovered ootaxa, particularly in Elongatoolithidae, with transitional morphologies. Other terms cited in describing ornamentation styles include ‘reticulate’ (*Paraelongatoolithus* (Qiang et al. 2010)), ‘nodular’ (*Macroelongatoolithus* (Wang et al. 2010)), ‘grainy’ (*Macroelongatoolithus* (Kim et al. 2011)), ‘bifurcating’ (IVPP V20182–V20184; (Wang et al. 2016)), ‘striated’ (*Protoceratopsidovum* (Choi et al. 2022)); and ‘verrucous’ (*Macroolithus* (Xing et al. 2023)), among a plethora of others. Frequently, the colossal elongatoolithid *Macroelongatoolithus* is described with three of the ornamentation styles on a single egg (Huh et al. 2014; Simon et al. 2018; Xing‐sheng et al. 2007), which is problematic for diagnosing ornamentation even for whole eggs, and exacerbated when considering only eggshell fragments (Zelenitsky et al. 2000; Hedge et al. 2024). In this study, we present a statistically rigorous methodology for analyzing and comparing the surface morphology of eggs that have external eggshell ornamentation. By ‘ground‐truthing’ this methodology in the extant eggs of *D. novaehollandiae* , we aim to test whether our methodology can quantitatively distinguish differences noticeable to the naked eye and detect external ornamentation variation between extant and fossil eggs of comparable avian and non‐avian dinosaurs. Our aim is to quantify ornamentation, add granularity to and remove subjectivity from categorizations of eggshell ornamentation used in parataxonomy, and empower studies of underlying behavioral aspects of egg‐laying in these taxa where ornamentation exhibits the most extreme variation.

**TABLE 1:** Current ornamentation categories and their definitions, after Carpenter (1999).

Term	Definition
Compactituberculate	Rounded nodes extending from individual shell units
Sagenotuberculate	Reticulate ridges with pits and grooves
Anastamotuberculate	Branching, wavy or anastomosing ridges
Linear(i)tuberculate	Chains of ridges and nodes
Ramotuberculate	Irregular chains of nodes and short ridges
Dispersituberculate	Scattered isolated nodes

## 2 Materials and Methods

Data for our extant taxon were collected during a week‐long excursion to Amaroo Hills Emu Farm in Guilford County, North Carolina in January 2022, during the end of the annual laying season. We selected eggs from the smallest subset of the domestic population that best simulated an authentic analogue for a polygynandrous *D. novaehollandiae* laying system in the wild (*n* = 23, at least six different females (Taylor et al. 2000)). We observed egg‐laying behavior during the hours around dusk remotely using two GoPro Hero 11 cameras from the NCSU libraries, and collected newly laid eggs (*n* = 6) at the end of each day. To ascertain relationships between eggs and the individual laying them, females were tagged with non‐toxic colored spray paint.

Our fossil data comes from the partially preserved clutch of an oviraptorosaur dinosaur, NCSM 33576. The clutch preserves a total of 16 eggs. We used the best‐preserved eggs (*n* = 8) from the underside for this study. No associated osteological remains are preserved, but there is a well‐established association between oviraptorosaurs and the ootaxon Elongatoolithidae in the fossil record (Mikhailov et al. 1996; Norell et al. 1994; Varricchio et al. 2001), and undescribed osteological material that is referrable to an appropriately proportioned oviraptorosaur from the Mussentuchit Member is currently under study (Makovicky 2014, 2023). The eggs of NCSM 33576 are laid in pairs, a trait typical of the monoautochronic ovulation exhibited by this clade (Varricchio and Jackson 2016). NCSM 33576 was recovered from the ‘Deep Eddy’ locality in the Mussentuchit Member of the Cedar Mountain Formation, an early Cenomanian‐age unit (Tucker et al. 2022) representing a near‐marine paleoenvironment in paralic succession across Central and eastern Utah.

**FIGURE 2:**
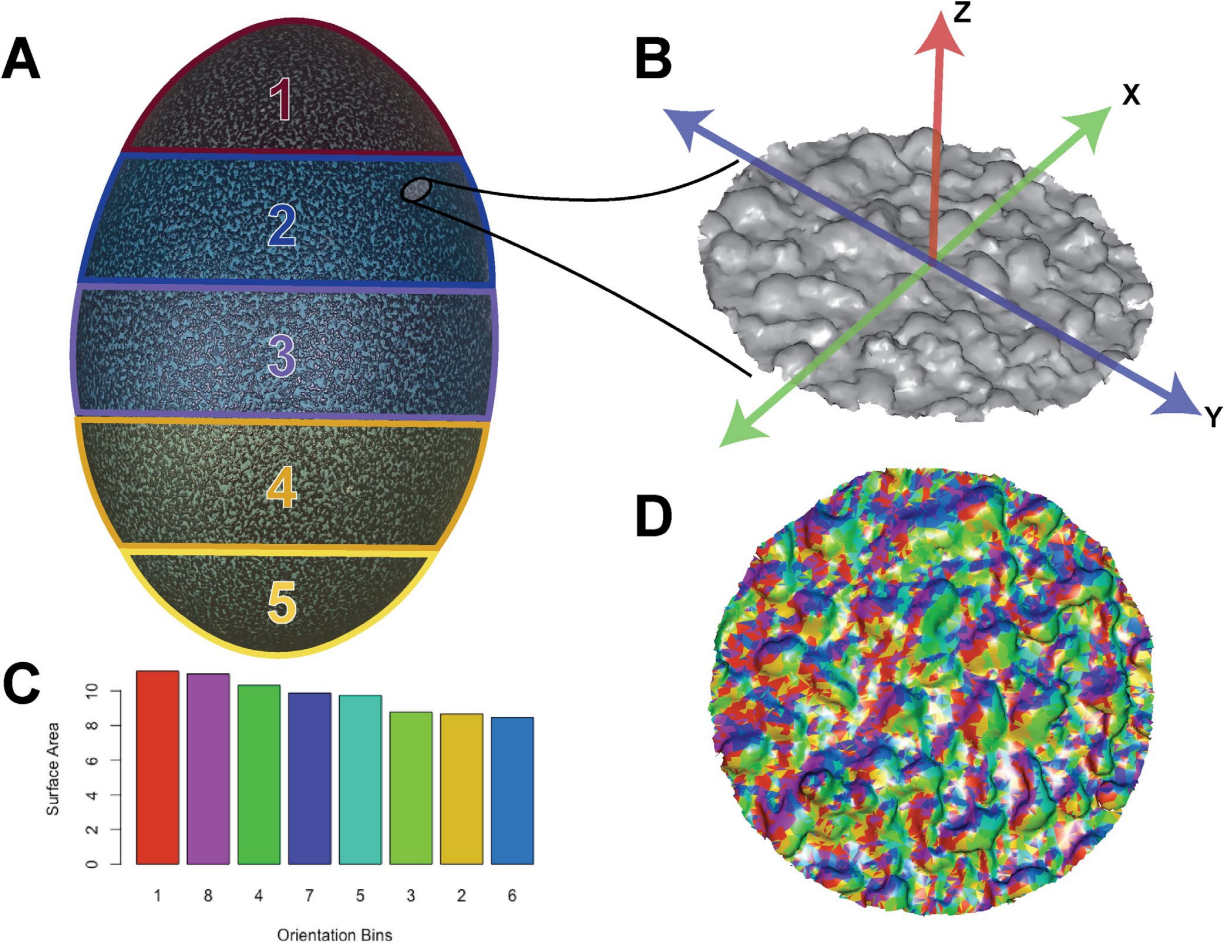
Graphical representation of methodological workflow for the statistical analyses in this study. (A) Zones 1–5 across the *Dasyornis novaehollandiae* egg NCSM 22850, following the method of Simon. Inset, a 1 mm radius surface subsample scan created using Metascan and Blender. (B) Extracted surface mesh exported and aligned along the X‐Y axis in MeshLab. (C) The surface area of the surface mesh binned into the eight directions in the orientation patch count function in molaR. (D) Visual representation of the orientation directions from the OPC3D function.

To compare eggs, we measured morphometrics (egg length and width) using digital calipers. We weighed *D. novaehollandiae* eggs using digital scales and calculated egg volume using the equation from Hoyt (1979). We digitized the eight fossil and six extant eggs using the application Metascan on an Apple iPhone 12 Pro, following the methods of Avrahami and Herzog (2023). We coupled the iPhone camera with a 15x macro lens and a 10 cm diameter ring light. Meshes built this way achieved comparable resolutions to μCT scans (mean pixel size of *c*. 11 μm) and provided a methodology that could compare *D. novaehollandiae* eggs with *M. carlylei* that could not be CT scanned at high resolution in its current nest configuration. These high‐fidelity scans also compare favorably to other portable surface scanners for the relatively low relief and high detail of eggshell surface texture versus other paleontological surfaces. Completed scans were exported as .gltf files from the application, preserving all raw data including texture and coloration. These files were imported to Blender (version 3.4). Here, we extracted uniformly sized (10 mm diameter) sections from the scanned eggshell surfaces. We collected as many of these as possible per egg while avoiding the effects of taphonomic cracks or non‐representative breaks on the surface of the eggs that could alter their original surface topography. Eggs were included in the study only if at least 30 sections could be obtained. We extracted surface sections across the eggs by adding 10 mm diameter cylindrical meshes through the surface and using the Boolean modifier to intersect cylinders with the egg surface (Figure 2A). The sections were exported as .stl files using the batch export feature and were subsequently imported into MeshLab2022.02 where they were aligned on the X‐Y axis (i.e., with a vertical Z axis; Figure 2B) and decimated to a standard 5000 triangular faces using the Quadric Edge Collapse Decimation tool. To import these into R for analysis, we exported aligned surfaces as .ply files. We binned the samples from each egg into one of five equal‐length zones along the long axis of the egg (Figure 2A), consistent with those described in (Simon) and matching egg position as they were laid *in nidus*.

To quantitatively analyze the topography of each surface, we used molaR (version 5.3) (Pampush et al. 2016), an R package with a suite of functions developed initially for topographical analysis of dentition based on 3D surface meshes, introducing quantitative metrics and eliminating the need for homologous landmarks. Each .ply mesh was analyzed using two of the functions existing in molaR: Dirichlet Normal Energy (DNE) (Bunn et al. 2011) and Slope (Dennis et al. 2004). These two metrics provide proxies for surface complexity and relief respectively. DNE measures curvature and undulation via an integral equation that sums the multiplied dirichlet energy densities of all faces on a mesh (a full explanation is provided in (Bunn et al. 2011)). Slope calculates the average relief of the triangles on the mesh (Dennis et al. 2004). We also modified the Orientation Patch Count function (Evans et al. 2007) to write a new function called Orientation, which analyzes the relative surface area of faces occupying each of the eight 45 directional bins (Figure 2C) to calculate both the direction and strength of orientation (pole‐to‐pole along the egg long axis or around the egg short axis; full details below). Each egg was assessed for normality in advance using the Shapiro–Wilk normality test, and found to have non‐normal distributions of complexity, relief and orientation. Outliers were removed using the interquartile range (IQR) with a greater multiplier on the upper bound to account for right‐skewed data. 95% confidence intervals of the mean differences in metrics were computed using bootstrapping with 1000 iterations. We used exact two sample Kolmogrov‐Smirnov tests to test the statistical significance of differences between distributions of *D. novaehollandiae* and *M. carlylei* eggs, which were both non‐normal and of different lengths. For each metric, we used both full datasets and datasets averaged per egg zone to reduce the likelihood of sampling size bias. Mean values for DNE, Slope, and Orientation are reported for each zone to represent trends across the egg, as an average of sub‐sampling within these zones that was intended only to give uniform, unaltered meshes. We calculated the inter‐relationship of metrics using Spearman's Rank correlation coefficient. We calculated harmonic mean *p*‐values from the R package ‘Pracma’ (Borchers and Borchers 2019; Wilson 2019) to meta‐analyze the *p*‐values found for the three metrics. We selected a harmonic mean over a meta‐analysis like Fisher's method (Wilson 2019) to account for the non‐independence of DNE, Slope, and Orientation values.

### 2.1 Orientation

Orientation is a new function in the molaR package (Pampush et al. 2016) that draws on the existing Orientation Patch Count function (OPC). The existing OPC function calculates the number of patches (where a patch is three or more triangles on a 3D mesh) that fall into each 45° bin about the X‐Y plane (Figure 2D). This measure alone does not distinguish patch count size because a patch can incorporate three adjoining faces but also orders of magnitude more faces, and these are calculated by OPC as a single patch of equal value. To account for directionality, we use the surface area value from the ‘Patch Details’ output of this function, which calculates the total surface area occupied by faces in each bin. The function then calculates the ratio of surface area in the bins oriented along the long axis (bins 1, 4, 5 and 8) to those oriented around the short axis (bins 2, 3, 6, 7). An additional calculation is added on to this ratio to produce a scale from −100 to +100 that indicates both the direction (positive along the long axis; negative around the short axis) and strength (higher numbers correlate to stronger directionality) of the orientation. This function will be available in the next update of the molaR package. We include new functions both for directionality of individual meshes (Orientation) and a batch export to a .csv file with all three calculated metrics, Surface Complexity, Orientation and Relief of Eggshell (SCORE). The scripts for both are provided in the Supporting Information.

## 3 Results

Quantitative ornamentation values are represented by three independent metrics from three functions that come directly or indirectly from the molaR package in R: these values represent different aspects of the surface topology that combined quantify the surface morphology of a given eggshell. Complexity is the value taken from the DNE function; Relief is taken from the Slope function; and Orientation is taken from a new function—Orientation—developed for this study that modifies the existing OPC function in molaR and provides a value for the overall orientation of the surface. The results, averaged for each egg, are provided in Table 2. Results averaged per egg zone are provided in Table 3. Full datasets can be found in the Supporting Information. Values are given to two decimal places. We consider *p*‐values to be significantly different when α < 0.05.

**TABLE 2:** Mean results for DNE, slope, orientation across *D. novaehollandiae* and *M. carlylei*.

	Pair	Egg	Complexity	Relief	Orientation		Egg	Complexity	Relief	Orientation
*M. carlylei*	1	1	53.88	12.49	1.12	*D. novaehollandiae*	1	15.21	6.63	12.61
	2	2	41.73	12.80	0.42		2	37.35	8.57	7.75
	3	3	32.80	10.93	1.66		3	47.40	8.72	9.61
	4	4	33.03	10.13	3.19		4	14.21	6.35	12.20
		5	37.21	10.39	8.11		5	33.29	8.35	8.97
		6	38.79	11.70	5.56		6	9.70	5.95	11.73
		7	44.37	13.12	2.37					
		8	40.42	11.84	7.10					

**TABLE 3:** Mean values and value ranges for DNE, slope and orientation across the egg zones of *M. carlylei* and *D. novaehollandiae* .

Zone	Complexity mean	Complexity range	Relief mean	Relief range	Orientation mean	Orientation range
*M. carlylei*						
1	53.35	42.38—70.20	13.21	12.02–14.36	3.04	−0.31–10.40
2	41.58	22.23—55.95	11.77	9.13–13.76	3.43	−1.90–9.08
3	37.47	22.28–62.97	11.09	8.46–13.36	4.17	0.33–9.34
4	36.20	23.53–49.81	11.19	9.22–13.60	4.68	−1.39–14.52
5	35.72	26.10–52.05	11.33	9.40–13.65	3.59	−21.3–13.86
*D*. *novaehollandiae*						
1	26.95	10.17–53.54	8.06	6.36–10.35	5.26	3.08–8.31
2	20.45	7.17–46.96	6.59	4.90–7.96	14.56	3.07–22.25
3	35.33	13.35–76.93	7.92	6.14–10.52	11.83	7.24–16.75
4	21.99	7.33–33.20	7.01	5.14–8.71	12.51	8.61–19.93
5	26.26	5.96–47.48	7.56	5.47–9.31	8.24	7.15–11.49

Broadly, our results demonstrate that the egg surface of *D. novaehollandiae* and *M. carlylei*, when considered at this taxonomic level, are significantly different from each other in terms of complexity, relief and direction, and meta‐analysis of these three metrics combined. *D. novaehollandiae* eggs are, as a whole, less complex, lower relief and more directional than *M. carlylei*. On an egg‐to‐egg basis, we find that Slope is the metric that differentiates the two taxa most often, with significant differences found in every pairwise comparison except one. In DNE, more than three‐quarters of the pairwise comparisons were significantly different between taxa, and in Orientation, just below three‐quarters. Harmonic mean *p*‐values demonstrate highly significant differences between *D. novaehollandiae* and *M. carlylei*.

### 3.1 Ornamentation Complexity via DNE

**FIGURE 3:**
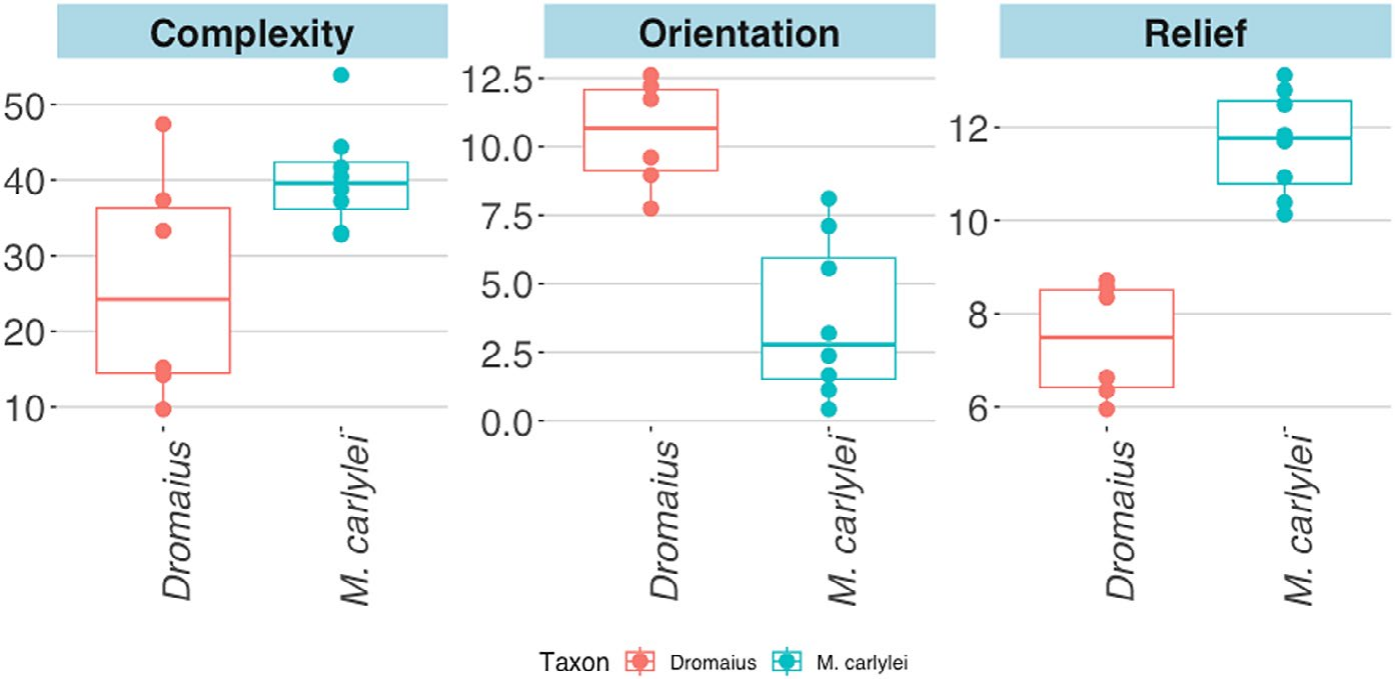
Boxplots of the ornamentation metrics for *D. novaehollandiae* (red) and *M.carlylei* (blue) eggs. Variation in (A) DNE, (B) orientation, (C) slope values. Corresponding numbers match the points on the graph and the images of the eggs.

Mean values for *D. novaehollandiae* and *M. carlylei* IQRs are 25.61 and 37.29 respectively, indicating greater complexity in our *M. carlylei* sample. The mean difference for the two taxa is estimated at 11.69 (95% CI: [9.01, 14.43]), and the *p*‐value for the difference in these means from the K‐S test is ≪ 0.01. Values for the complexity metric are highly variable from egg to egg across both *D. novaehollandiae* and *M. carlylei* egg samples (Figure 3A). Mean *D. novaehollandiae* DNE values for one egg range from 9.70 to 47.40, and *M. carlylei* from 32.80 to 53.88, suggesting greater whole‐egg variance between individual *D. novaehollandiae* eggs in our sample than between individual *M. carlylei* eggs. A K‐S test of the whole nest distributions between *D. novaehollandiae* eggs and *M. carlylei* demonstrates significant differences, with a *p*‐value ≪ 0.01.

Inter‐egg variation is also detected in DNE between eggs of the same taxon. Within *D. novaehollandiae* eggs, K‐S test *p*‐values are significant in 80% of comparisons. In *M. carlylei*, distributions are significantly different in 71% of comparisons. Of the 48 pairwise comparisons of individual eggs across both groups, 77% had significant differences.

**FIGURE 4:**
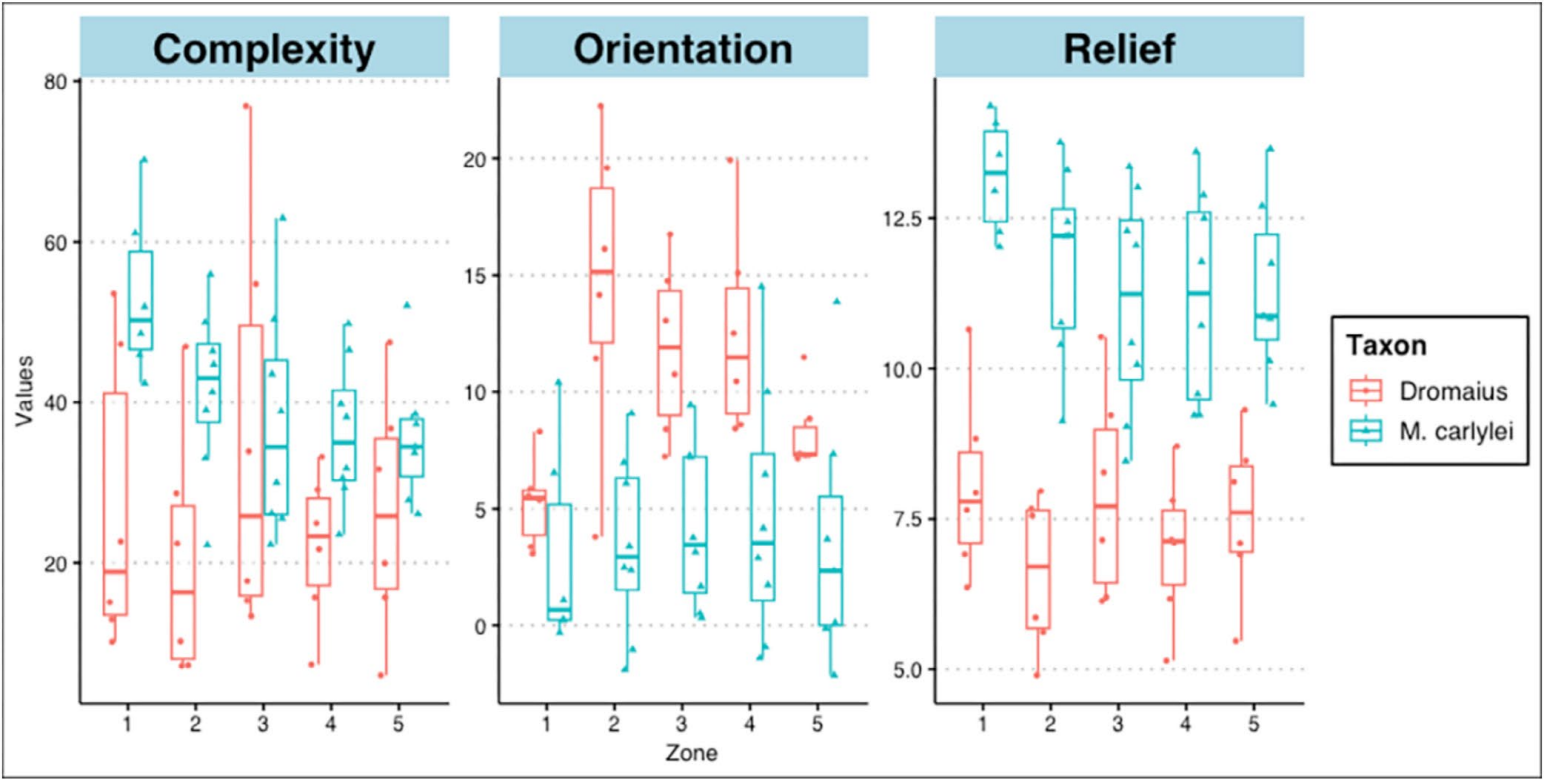
Intra‐egg variation across the egg zones of *D. novaehollandiae* (red) and *M. carlylei* (blue) eggs. Box plots for each egg showing (A) DNE, (B) orientation, (C) slope values.

Across individual eggs of *D. novaehollandiae* , mean DNE values are highest in Zone 3, followed by the two poles, Zone 1 and Zone 5, and lowest in the regions between the middle and the poles (Zone 4 and Zone 2). Patterns are different in the *M. carlylei* (Figure 4A), where DNE values decrease through the zones in ascending order: highest DNE in Zone 1, then Zone 2, Zone 3 and the lowest values in Zone 4 and Zone 5.

### 3.2 Topographical Relief via Slope

Mean slope values for IQRs of *D. novaehollandiae* and *M. carlylei* are 7.47 and 11.45 respectively, evidencing a higher relief metric for *M. carlylei*. The mean difference for the two taxa is estimated at 3.98 (95% CI: [3.67, 4.27]). The difference in distributions from a K‐S test is strongly significant (*p*‐value ≪ 0.01). Slope values for each *D. novaehollandiae* egg range from 5.95 to 8.72, whereas in *M. carlylei* the range is 10.13–13.12 (Figure 3B).

Slope value distributions are also variable within ootaxa. There are significant differences between 73% of the *D. novaehollandiae* eggs. In *M. carlylei*, there are significant differences between 71% of the pairwise comparisons. In comparisons of inter‐pair eggs of *M. carlylei*, significant differences are found in 96% of tests.

Within eggs, the relief is consistent between the different zones (Figure 4B). In *D. novaehollandiae* eggs, the highest relief is in Zone 1, then Zone 3, then the opposite pole Zone 5, Zone 4 and the lowest in Zone 2. In *M. carlylei*, the highest values are also in Zone 1, then decline in Zone 2, Zone 5, Zone 4 and the lowest in Zone 3.

### 3.3 Direction via Orientation

The mean values for orientation, the metric assessing direction, are 11.09 for *D. novaehollandiae* eggs and 4.09 for Deep Eddy. All mean values are > 0 when averaged for each egg zone, specifying direction that moves along the long axis rather than around the short axis, but some *M. carlylei* eggs have low negative values in certain zones, indicating a weak orientation around the short axis rather than along the long axis. The mean difference is estimated at 7.00 (95% CI: [5.77, 8.2], *p*‐value ≪ 0.01). K‐S tests of orientation distribution are highly significant between *D. novaehollandiae* and *M. carlylei* (*p*‐value ≪ 0.01). *D. novaehollandiae* eggs vary in average orientation from 7.75 to 12.61, whereas *M. carlylei* vary from 0.42 to 8.11 (Figure 3C).

Orientation values within ootaxa are more similar than for DNE and Slope. In *D. novaehollandiae* eggs, five (33%) have significant differences. In *M. carlylei*, 57% have significant differences. Comparing individual eggs between the ootaxa, 83% of pairwise comparisons had significant differences.

Across individual eggs of the *D. novaehollandiae* , mean orientation values were much higher in Zones 2, 3 and 4 than in the polar Zones 1 and 5 (Figure 4C). Zone 2 had the highest value, then Zone 4, Zone 3, Zone 5 and Zone 1 had the lowest. In *M. carlylei*, orientation values were much less variable between zones. The highest mean value was in Zone 4, followed by Zone 3, Zone 5, Zone 2 and the lowest in Zone 1.

### 3.4 Spearman's Rank Correlation Coefficient

We tested for correlation between the three metrics of *D. novaehollandiae* and *M. carlylei* egg ornamentation (Table 4) using non‐parametric Spearman's rank correlation coefficient (ρ), as our data were non‐normally distributed. In both *D. novaehollandiae* and *M. carlylei*, higher relief egg zones also tend to be more complex. For the *D. novaehollandiae* eggs, this is the middle zone of the egg, whereas in *M. carlylei* it is Zone 1 at the egg pole. Higher relief and higher complexity zones are also less directional. In *D. novaehollandiae* eggs, complexity and relief have a strong positive correlation (Spearman's ρ = 0.908). Both complexity and relief have negative correlations when tested against direction, but both of these metrics were weakly correlated. For complexity and direction, Spearman's ρ = −0.348 and for relief and direction, Spearman's ρ = −0.351. The trends are similar in *M. carlylei*, but the correlations are not as strong: complexity and relief ρ = 0.835; complexity and direction have almost no correlation (Spearman's ρ = −0.084); and relief and direction similarly have a weak Spearman's ρ (−0.138). When both *D. novaehollandiae* and *M. carlylei* are included, complexity and relief retain a strong correlation, though weaker than either taxon individually (ρ = 0.807). The correlation for complexity and direction (ρ = −0.233), and the correlation between relief and direction (ρ = −0.349), are stronger than in exclusively *M. carlylei*.

**TABLE 4:** Correlations between the three metrics in this study using Spearman's rank correlation coefficient (Spearman's ρ).

Correlation (Spearman's ρ)	*D. novaehollandiae*	*M. carlylei*	Both taxa
Complexity~Relief	0.908	0.835	0.807
Complexity~Direction	−0.348	−0.084	−0.233
Relief~Direction	−0.351	−0.138	−0.349

### 3.5 Harmonic Mean *p*‐Values

In addition to analyzing the relationships between the taxa, eggs and egg zones with individual DNE, Slope and Orientation metrics, we performed a meta‐analysis to combine the results of our tests using the harmonic mean *p*‐value (Borchers and Borchers 2019; Wilson 2019).

**FIGURE 5:**
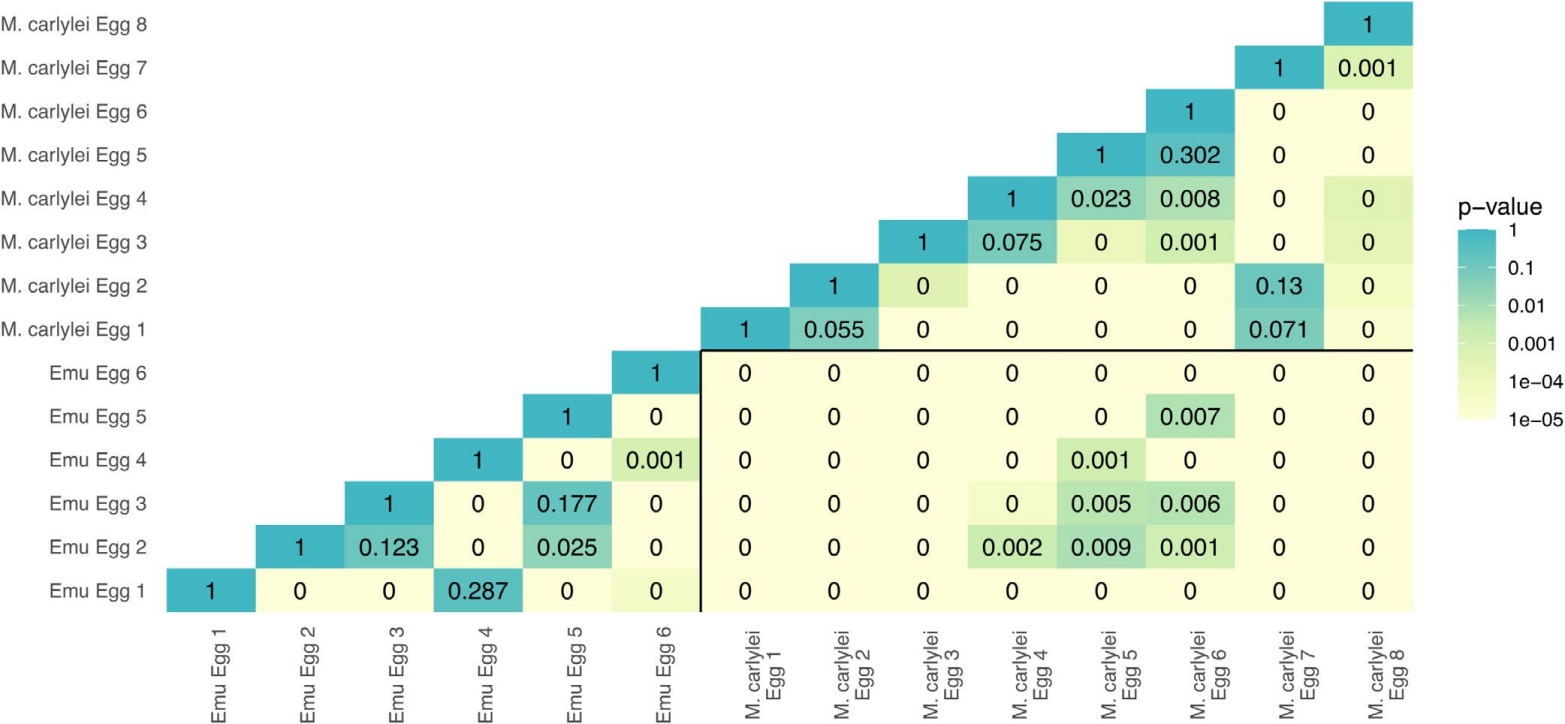
Heatmap of the harmonic mean *p*‐values between each egg included in the study. *D. novaehollandiae* and *M.carlylei* eggs are separated by black lines.

Harmonic mean *p*‐values for the combination of DNE, Slope and Orientation highlight that *D. novaehollandiae* eggs can be distinguished from each other in terms of their ornamentation (Figure 5). Of the 15 pairwise tests, 80% were significantly different. This pattern is somewhat similar when comparing within *M. carlylei*: 82% showed significant differences. Strikingly, the harmonic mean *p*‐values for all 48 pairwise tests of eggs from different taxa were significantly different. Pairwise comparison of whole taxa tested against each other similarly produced highly significant results (harmonic mean *p*‐value ≪ 0.01).

## 4 Discussion

Our results show that quantification of external eggshell ornamentation using DNE, Slope and Orientation can distinguish between the eggs of an extant avian ( *D. novaehollandiae* ) and an extinct non‐avian theropod dinosaur (*M*. *carlylei*). Whereas using previous methodology we were able to qualify these differences based on gross inspection, we can now numerically compare these taxa to statistically designate how different they are and explore variation in ornamentation at a granular level including across single eggs, within clutches and intraspecifically—a more powerful tool for directly testing hypotheses. The quantitative algorithms also corroborate the visual patterns we can see (i.e., eggs that look more complex, with greater relief and more directionality have higher corresponding DNE, Slope and Orientation values).

### 4.1 Assessment of Metric Variation

What is most apparent is the near‐total lack of overlap in the Slope distributions between *D. novaehollandiae* and *M*. *carlylei* eggs, with highly significant differences between the low‐relief *D. novaehollandiae* eggs and the high‐relief *M. carlylei*. This indicates that the relief of an eggshell's ornamentation can be quantifiably discretized and is potentially taxonomically useful. The convergence of ornamentation structures in *D. novaehollandiae* and *M. carlylei* is not homologous and differing slope values may reflect this. If so, we would predict similar Slope values between the eggs of other oviraptorosaur ootaxa (Elongatoolithidae), and perhaps more broadly in non‐avian dinosaurs, than to *D. novaehollandiae* eggs. The external layer in *D. novaehollandiae* forms prismatically over an organic porous matrix (‘tubule plexus’ (Board and Tullett 1975); ‘porous organic layer’ (Dauphin et al. 2006)), and this matrix is connected to the atmosphere by short pores that occupy low relief areas between the prisms (Board and Tullett 1975). Any space not occupied by pores is potential space for the prismatic external layer to coalesce. The process is poorly understood and warrants further investigation. Nonetheless, this differs from oviraptorosaur two‐layered eggshell, where ornamentation is formed by manipulation of the continuous layer. Both taxa are seemingly experiencing similar selection for ornamentation, but with different physiological formation constraints. Future work in this area could elucidate the taxon predictability and granularity of the trends in eggshell ornamentation relief.

*D. novaehollandiae* and *M. carlylei* eggs that look the most complex on gross inspection produce the highest DNE values. These eggs have more nodes and ridges creating external morphological patterns, and there is great variation between these eggs borne out in the data. DNE values vary as much or more for eggs of the same taxon as they do for eggs between taxa, suggesting that the mechanism controlling for mean eggshell complexity is somewhat decoupled from taxonomy. In *D. novaehollandiae* eggs, there is greater complexity variation across the eggshell than in *M. carlylei*.

Orientation values, like Slope, seem to reveal that directionality in this study is largely clade‐dependent, and the difference in mean values between *D. novaehollandiae* and *M. carlylei* supports this. However, there are eggs within a single taxon or ootaxon that do have similar (i.e., not statistically significantly different) ornamentation, and these patterns could be determined more systematically than stochastically. These non‐significant intraspecific tests are restricted to three *M. carlylei* eggs, two of which are paired and that have the highest orientation values within the nest. In *M. carlylei*, this ornamentation is easier to relate to the subjective categories that govern ootaxonomy. Eggs with a more linear tuberculate ornamentation pattern of nodes and ridges forming linear structures from pole to pole have a higher Orientation value than eggs with a more dispersed tuberculate pattern composed more of nodes than ridges.

Though not apparent on gross inspection, *D. novaehollandiae* eggs have a higher mean orientation than *M. carlylei*. The true directionality of their eggshell ornamentation is likely masked on gross inspection by a glossy appearance and lower eggshell relief. Orientation trends are somewhat easier to see in older *D. novaehollandiae* eggs where the blue‐to‐green pigmentation has dulled to dark gray (JH, pers. obvs.). *M*. *carlylei* eggs are highly variable in direction values: some eggs of NCSM 33576 exhibit clear ornamentation direction, and values for these eggs are more comparable in Orientation to the *D. novaehollandiae* eggs. Distributions are not as different as in Slope, but mean values do not overlap like with relief, perhaps suggesting some clade‐based differentiation. Directionality decreases sharply in the *D. novaehollandiae* eggs toward the poles, whereas in *M. carlylei* the trend is much more evenly distributed across zones. This looks to be dictated by egg shape: *D. novaehollandiae* eggs are much wider in Zone 3, thus the direction of the nodes and ridges are constrained toward a more confined area when tapering to the poles. Oviraptorosaur eggs, named elongatoolithids for their elongated shape, have markedly less tapered poles and the orientation values in the eggs we measured do not exhibit a similar drop off in Zones 1 and 5.

We caution that high direction values were seen in *D. novaehollandiae* eggs with particularly low slope values, and it could be that the Orientation function performs less effectively on lower relief surfaces. Further, the area size of the sample may also affect this function. Smaller sections relative to orientation size have the potential to truncate linear ornamentation, and this could potentially reduce signal. Researchers should use the largest sections possible for calculating orientation metrics within the framework of zone delineation and taphonomic alteration. In our study, we increased the section size from an initial 5 mm diameter to a 10 mm diameter section to account for the potential of truncation bias. By testing for the harmonic means of the *p*‐values for each metric, we could assess the combined effects of DNE, Slope and Orientation on overall ornamentation distribution between *D. novaehollandiae* and *M. carlylei*. The prominence of significant differences between *D. novaehollandiae* and *M. carlylei*, particularly in Slope and Orientation values, was consistent with harmonic mean *p*‐values between taxa.

### 4.2 Elongatoolithid Ootaxonomy Implications

**FIGURE 6:**
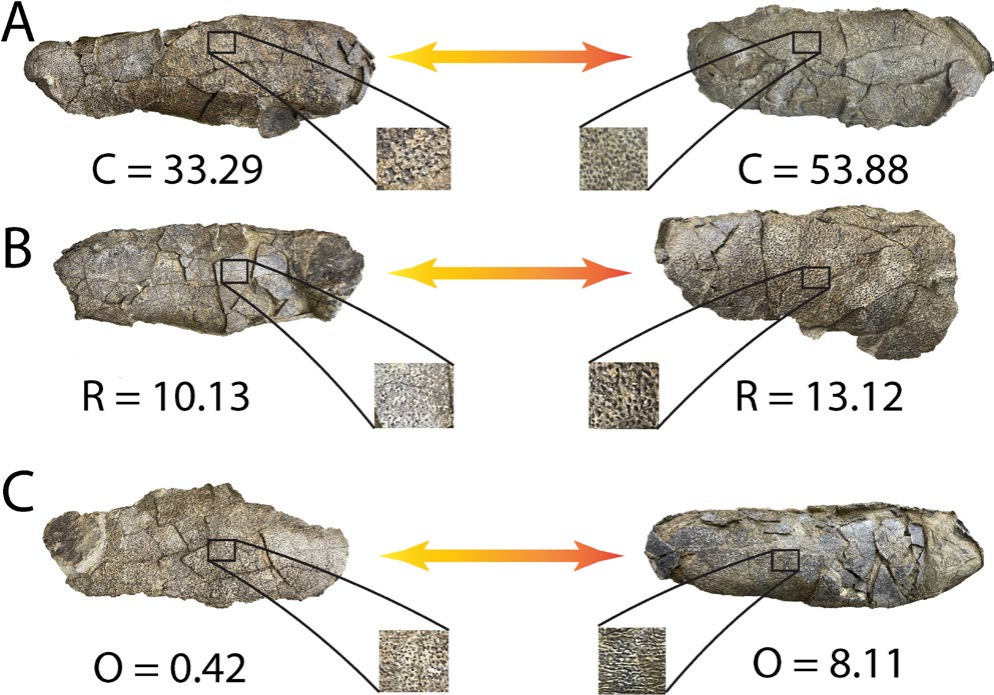
Comparisons of visual and numerical ornamentation metrics within the *M. carlylei* nest NCSM 33576 (A–C).

Our intent here is not necessarily to replace the well‐established ornamentation categories in ootaxonomy, which are entrenched in the literature and have intrinsic qualitative value, if applied consistently. Moreover, these statistical values are complementary to the categories and do not directly correlate to any one of them. Nonetheless, we do offer a proof of concept herein for a method that significantly updates these categories that could be used in tandem. We suggest reporting in new studies not only categories of ornamentation for eggs as previously, but combining these with numerical values of complexity, relief and direction. The statistical values add resolution to these categories, offering tangible, updated descriptions of the ornamentation that assist in discerning more granular differences. This is especially beneficial to the eggs of Elongatoolitihidae, where variation in ornamentation types can now be supported by numerical values to better differentiate and diagnose ootaxa. Previous descriptions of ornamentation for nodes and ridges have been expanded in the literature into a plethora of terms (see Introduction), along with the frequent division into coarser (Huh et al. 2014; Simon et al. 2018; Zelenitsky et al. 2000) and finer nodes and ridges (Wang et al. 2016; Grellet‐Tinner et al. 2006), and can now be better refined. DNE provides this, and the ‘coarser’ looking egg ornamentation corresponds in our data with higher DNE values (Figure 6A). Our results are consistent with that of Yang and Sander (2022) who report that the acute end (Zone 1) are more ornamented than the blunt end (Zone 5) in Elongatoolithidae.

Orientation values have long been recognized as characteristic of Elongatoolithidae, in particular as lineartuberculate or linearituberculate (Simon et al. 2018; Carpenter 1999; Kim et al. 2011; Taylor et al. 2000; Mikhailov 1991; Dong and Currie 1996; Pu et al. 2017; Norell et al. 2018; Bi et al. 2021). These have been informally divided into ‘weak’ orientation (Liang et al. 2009) versus terms like ‘densely‐packed’ (Xing et al. 2020), as well as into categories that cross multiple shell units versus individual shell units and subunits (Mikhailov 1991) and those that are oriented and unoriented (Hedge et al. 2023); sensu Carpenter (1999). Terminology describing categories of ornamentation is subjective, and Orientation values herein provide a quantitative framework for assessing the direction and strength of ornamentation. Ornamentation in Elongatooltihidae eggshell, and *D. novaehollandiae* as described in this study, is almost always pole‐to‐pole, and Orientation analysis reflects this with positive values. We add granularity to this, with larger positive values indicative of ‘stronger’ pole‐to‐pole orientation. Previous diagnoses and descriptions of the colossal ootaxon *Macroelongatoolithus* (Huh et al. 2014; Simon et al. 2018; Pu et al. 2017) have suggested that ornamentation is lineartuberculate at the equator with a gradation toward dispersituberculate or smooth ornamentation at the poles, although one pole may be more ornamented than the other (Yang and Martin 2022). Results in this study (Table 1) suggest that this may not always be the case, and that variation in pole‐to‐pole orientation of ornamentation may be more granular. Our results demonstrate that this pattern of variation is inconsistent between different eggs, particularly between pairs, where eggshell orientation is more variable than between different zones (Figure 6C). Some of our eggs exhibit lineartuberculate ornamentation at the equator, but other pairs have a more dispersituberculate or ramotuberculate ornamentation across the whole egg, more consistent with the diagnosis in Zelenitsky et al. (2000) where ornamentation in the oogenus is restricted to ‘variable’. Previously, description of this nest would be restricted to ‘variable’ when looking at the whole clutch, or that some pairs look dispersituberculate while others are lineartuberculate. Reporting complexity, relief and direction values could allow some distinction to be made between similar looking ootaxa, and test underlying hypotheses of variaiton. Future studies across multiple clades could consider using the numerical scale of directionality to define the boundaries of ornamentation categories.

Slope values are indicative of variable relief, intrinsic to ornamentation but previously were not assessed as part of the categorization of ornamentation. Elongatoolithid eggs among theropods have dramatic variation in their ornamentation (Figure 6B); the eggs of *Undulatoolithus pengi*, for example, are diagnosable by their extremely tall ornamentation, which can be up to half of their total eggshell thickness (Zelenitsky et al. 2000; Wang et al. 2013), but categorically there has been no way to assess this within the ootaxonomic framework. Further work into quantitative ornamentation could reveal these phenomena using non‐destructive methods.

### 4.3 Quantifying *Dromaius novaehollandiae* Eggshell Ornamentation

Eggshell ornamentation in *D. novaehollandiae* has been noted in the literature (Board and Tullett 1975; Szczerbińska and Wiercińska 2014, 2010; Tullett 1984; Panheleux et al. 1999) and compared to eggs of other extant Palaeognathae and extinct non‐avian dinosaurs (Grellet‐Tinner 2006; Mikhailov 1997; Choi et al. 2023) but the variation across the egg and between eggs has to our knowledge not been reported nor described. In the *D. novaehollandiae* eggs from this study, this variation is clear visually, and differences have now been revealed statistically. Our eggs were collected from an emu farm in North Carolina, where the laying season ranges from mid‐November to early January, but can be as long as 188 days in other populations (Szczerbińska et al. 2014). Although we marked female individuals as they were laying, the lateness of the laying season meant that we collected no more than a single egg from any single individual. Moreover, eggs were laid in various corners of the paddock as surrogate nests, with four of the six eggs laid in a single ‘nest’ and one egg each in two other ‘nests’. We cannot compare the eggs within an individual to assess variation, but the non‐significant differences between some of the eggs suggest that variation occurs above the individual level. As discussed above, *D. novaehollandiae* eggs are known to differ in size in correspondence with the age of the laying hen (Szczerbińska et al. 2014; Quintero et al. 2022). The differences seen between groups of *D. novaehollandiae* of 2 and 7 years in age in Quintero et al. (2022) demonstrated increases in egg length, width and weight in the older group (although a non‐significant increase in egg volume due to the change of egg shape). However, in our sample, where the age of the hens was unknown, there is no strong correlation between egg length and egg width (*R* ^2^ = 0.365) or egg length and egg mass (*R* ^2^ = 0.022). Egg lengths and widths are similar in eggs that have non‐significant differences in harmonic mean *p*‐values (NCSM 22852, length = 136.8 mm and NCSM 22854, length = 137.0 mm; NCSM 22851 and NCSM 22852, width = 91.9 mm), but this trend is not consistent across all our eggs with non‐significantly different eggshell ornamentation. Further, eggs NCSM 22850, NCSM 22851, NCSM 22852 and NCSM 22854 were laid in the same ‘nest’, suggesting no in‐nest ornamentation differentiation as some of these eggs were indiscernible statistically, although the proclivity of hens in the paddock to lay only in paddock corners does limit the efficacy of this conclusion. Both this study (*n* = 6) and that of Quintero et al. (2022) (*n* = 9) have low sample sizes that are likely not representative of true trends, and we suggest future research into larger scale trends of egg morphometrics and corresponding ornamentation patterns.

### 4.4 Future Applications of SCORE

The methodology we describe in this paper uses a new method from the molaR package in R (Simon). By adding a new function (Orientation) that quantifies directionality, we have provided a three‐proxy framework for assessing eggshell surfaces. Our second new function, SCORE, outputs all of these metrics together for further analysis. However, we want to highlight that this methodology should not be restricted to only applications of eggshell surfaces. The molaR package, as the name suggests, was developed for quantifying dentition surfaces (Simon), but even this initial publication promotes its use on ‘other topographic surfaces’ in its title. The molaR package has been used to study the dentition of mammals (Pampush et al. 2019, 2022; López‐Aguirre et al. 2022; Villalobos‐Chaves and Santana 2022), other amniotes (Melstrom and Wistort 2021; Shipps et al. 2023; DeMers and Hunter 2024) and conodonts (Kelz et al. 2023), as well as in turtle shell patterning (Pamfilie et al. 2023). To our knowledge, this is the first novel application of molaR for eggshell surfaces. We believe the addition of the directionality proxy adds powerful additional quantitative information that promotes greater use of molaR in existing applications, to eggshell surfaces as in this study and more widely in the quantification of paleotopographic surfaces. Moreover, our initial method for surface scanning specimens has been calibrated to be a highly efficient, accurate and portable technique for high‐resolution scans of these surfaces.

## 5 Conclusion

In this study, we have established that visible differences in eggshell ornamentation can be corroborated with quantitative evidence, both in the external eggshell morphology between extant avians and non‐avian theropods, but also at an extra layer of granularity between the eggs of the same taxon, and even within the same nest. By making novel use of the molaR package in R (Pampush et al. 2016), we establish a three‐proxy system for quantifying eggshell ornamentation using existing metrics for relief (Slope) and complexity (DNE), as well as a new function presented in this study as a proxy for directionality (Orientation). Thus, we have created the first quantitative framework for better‐assessing differences between different oviraptorosaur dinosaur eggs, facilitating a much‐needed improvement on the existing categorization of this eggshell characteristic. This will be vital in the ongoing understanding and interpretation of eggshell ornamentation, particularly in non‐avian dinosaurs like oviraptorosaurs where the ornamentation is at its most diverse. Avenues of research into the underlying ecological and evolutionary drivers of eggshell ornamentation can now be examined more rigorously. Further, we actively encourage that this technique be applied beyond eggshell ornamentation. Techniques in this study were adopted and adapted from existing research in fossil dentition (Simon; Dennis et al. 2004), and we have demonstrated their efficacy beyond this scope. We similarly extend an invitation to use and modify our techniques as a non‐destructive, inexpensive and efficient method of topographical analysis for any paleobiological surface.

## Author Contributions

**Joshua Hedge:** conceptualization (equal), formal analysis (lead), investigation (lead), methodology (equal), software (equal), validation (supporting), visualization (lead), writing – original draft (lead), writing – review and editing (equal). **Emerald Bender:** conceptualization (supporting), formal analysis (supporting), investigation (supporting), methodology (equal), software (equal), validation (equal), visualization (supporting), writing – original draft (supporting), writing – review and editing (equal). **Lindsay E. Zanno:** conceptualization (equal), formal analysis (supporting), funding acquisition (lead), investigation (supporting), methodology (supporting), project administration (lead), resources (lead), supervision (lead), validation (lead), visualization (supporting), writing – original draft (supporting), writing – review and editing (equal).

## Conflicts of Interest

The authors declare no conflicts of interest.

## Data Availability

The data that support the findings of this study are openly available in Dryad at https://doi.org/10.5061/dryad.70rxwdc6q.
